# Social Network Analysis Predicts Health Behaviours and Self-Reported Health in African Villages

**DOI:** 10.1371/journal.pone.0103500

**Published:** 2014-07-29

**Authors:** Goylette F. Chami, Sebastian E. Ahnert, Maarten J. Voors, Andreas A. Kontoleon

**Affiliations:** 1 Department of Land Economy, University of Cambridge, Cambridge, United Kingdom; 2 Department of Pathology, University of Cambridge, Cambridge, United Kingdom; 3 Theory of Condensed Matter Group, Cavendish Laboratory, University of Cambridge, Cambridge, United Kingdom; 4 Development Economics Group, Wageningen University, Wageningen, the Netherlands; Centre de Physique Théorique, France

## Abstract

The provision of healthcare in rural African communities is a highly complex and largely unsolved problem. Two main difficulties are the identification of individuals that are most likely affected by disease and the prediction of responses to health interventions. Social networks have been shown to capture health outcomes in a variety of contexts. Yet, it is an open question as to what extent social network analysis can identify and distinguish among households that are most likely to report poor health and those most likely to respond to positive behavioural influences. We use data from seven highly remote, post-conflict villages in Liberia and compare two prominent network measures: in-degree and betweenness. We define in-degree as the frequency in which members from one household are named by another household as a friends. Betweenness is defined as the proportion of shortest friendship paths between any two households in a network that traverses a particular household. We find that in-degree explains the number of ill family members, whereas betweenness explains engagement in preventative health. In-degree and betweenness independently explained self-reported health and behaviour, respectively. Further, we find that betweenness predicts susceptibility to, instead of influence over, good health behaviours. The results suggest that targeting households based on network measures rather than health status may be effective for promoting the uptake of health interventions in rural poor villages.

## Introduction

Infectious diseases remain a leading cause of morbidity and mortality in developing countries. Over one billion people, mostly in rural areas, are currently afflicted with one or more communicable diseases.[1]–[5] Many of these maladies are preventable with access to safe water, sanitation, and healthcare.[6] However, identification of people that are ill is a strenuous task in rural poor areas where access to formal medical care is scarce and infectious diseases are chronic.[7] Furthermore, behavioural modification to improve preventative health, such as the persuasion of households to use protected instead of open water sources and to use pit latrines instead of engaging in open defecation, proves challenging in practice.[8] Monitoring such behaviours is difficult, in particular for open defecation, as many of these behaviours are conducted in private. Therefore, indirect indicators, such as social popularity and influence, may offer a useful alternative to identify who is ill and whom to target for behavioural health interventions [9].

Social networks have been widely studied for understanding peer effects and the spread of behaviours in a variety of contexts.[10]–[15] The study of complex networks aims to provide insight into the connectivity of physical, natural, or social systems.[16] Two commonly examined network properties are degree and betweenness. The degree of an individual, or node, is the number of incoming and outgoing connections. Of primary focus for social networks is in-degree, the number of incoming connections.[17] In-degree conveys the popularity of individuals, by counting the number of people that have named that person as a friend. Betweenness is the proportion of shortest paths (here, a set of lines connecting two households) in a network that traverses the node of interest.[17] In contrast to in-degree, betweenness is a global measure of the network. High betweenness commonly is viewed as an indicator of information spreading in social networks.[18] However, it is an open question as to what extent betweenness and in-degree are important factors in determining self-reported physical health and actual health behaviours. These centrality indicators are particularly important in distinguishing how social placement affects health, as households that traverse the most paths connecting other households (high betweenness) need not have many friends (high in-degree) [19].

Existing studies [20]–[23] on social networks and health identified the dependencies between dyads of people in single non-interacting networks and showed that individuals were connected with other individuals of similar physical health. For example, Christakis et al. [22] and Liljeros et al. [23] found that high degree nodes were more likely to be sick or infected than low degree nodes. We expand upon this literature and study a set of remote villages in rural Liberia. Liberia is recovering from a civil war, which resulted in high levels of mortality and morbidity and broke down the delivery of basic health services. While important steps have been taken to rebuild the country, Liberia still ranks amongst the lowest in the world with respect to health indicators. Under-five mortality is 110 out of every 1000, the childhood malaria rate is 32%, reduced growth in children (stunting) is 42%, and access to formal health services is below 40% in remote areas.[24] We demonstrate the ability of social network indicators to predict health outcomes and to explain the susceptibility of a household to partake in health interventions in one of the most impoverished and under-researched developing country contexts. Especially, we find that in-degree predicts household physical health status and betweenness identifies individuals susceptible to positive behavioural influence and likely to respond to health interventions.

## Methods

### Ethical approval

We obtained verbal informed consent from the respondents. Research assistants went through an explanation of the research and asked respondents if they understood and agreed to go ahead. Respondents were informed that they were not obliged to answer questions if they did not want to and were free to stop the interview at all times. In rural Liberia very few people can read or write. The research assistant recorded the answer of the respondent and any remarks made on the survey form. We obtained Institutional Review Board (IRB) approval for this consent procedure and a Gola Forest study that encompasses the trans-boundary area of Sierra Leone and Liberia under the IRB of the University of Chicago, number H10076.

### Data sample and network construction

**Figure 1:**
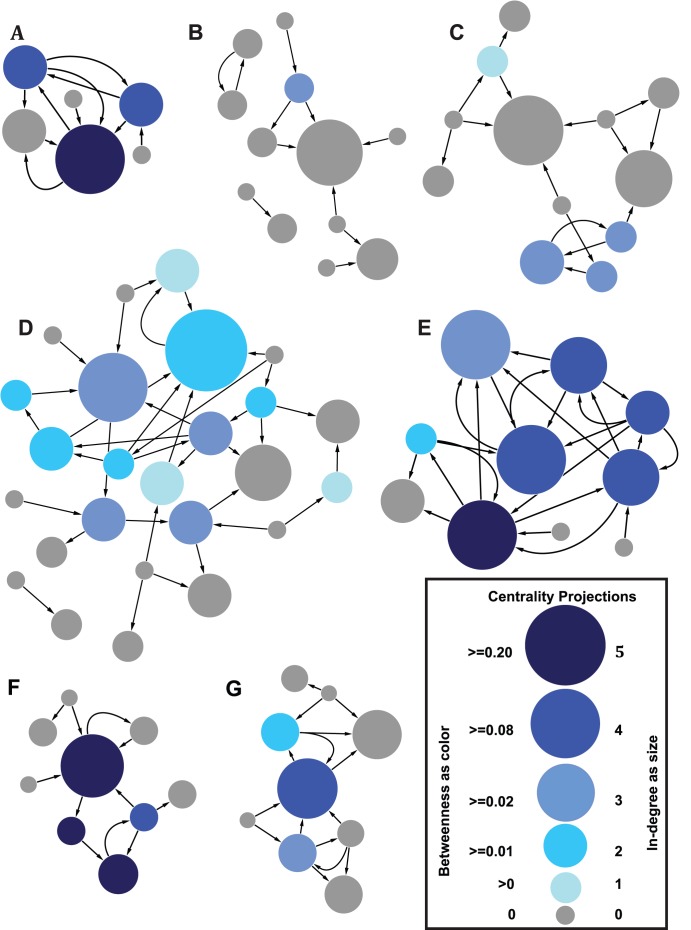
Friendship networks of 7 Liberian villages. Each village is labelled with an uppercase letter. **A)** Village 26007 with 6 nodes and 10 edges. **B)** Village 13233 with 12 nodes and 11 edges. **C)** Village 26036 with 12 nodes and 16 edges. **D)** Village 26016 with 25 nodes and 37 edges. **E)** Village 13247 with 10 nodes and 23 edges. **F)** Village 13111 with 9 nodes and 11 edges. **G)** Village 13245 with 9 nodes and 16 edges. There are a total of 83 nodes and 124 edges. The nodes represent households. The size of the node represents in-degree, which is the number of other households in the village that named the household of interest. The number of nodes and their in-degree in parentheses are: 23(0), 26(1), 19(2), 5(3), 9(4), and 1(5). The colour of the node represents betweenness centrality. In all networks, and particularly in **B)** and **C)**, not all nodes with high in-degree have high betweenness centrality. The directed edges (arcs) represent pairwise friendship connections between any two members of the households. An outgoing edge indicates that the sending household named the receiving household as a friend and vice versa. The curved edges in the graph show two households where there is reciprocity in friendship connections. If there were multiple edges between the same pair of households we treated them as one edge. The number of households and their betweenness range in parentheses are: 49(0), 4(<0.01 and > = 0), 7(<0.02 and > = 0.01), 10(<0.08 and > = 0.02), 7(<0.20 and > = 0.08), and 6(> = 0.20). See Table S3 in File S1 for network construction statistics.

During February – March 2012, we surveyed friendship networks in seven highly remote villages in post-conflict Liberia (Figure 1). One week in advance of survey activities, runners were sent to each village with a letter of invitation for the village chief. The runner reviewed the letter in detail with the chief, explained the proposed date of arrival, number of enumerators to be hosted, and a summary of proposed activities. Permission was requested from the chief for activities to be undertaken as planned. If permission was denied, the runner sought to determine whether an alternative date was available. If the chief refused the research team to visit at all, the runner informed the project leader immediately and a new village was selected from within the same geographical quadrant; however, refusal to conduct research in the village never occurred. Scheduled activities only were undertaken with permission from the chief. After securing approval to carry out the program, a project leader walked around the village and created a numbered household list, which contained names of all household heads.

The full household list was used in a public lottery to randomly select households. Paper slips with household numbers were placed in an opaque bag. During a public meeting of all households in the village, a child was invited to draw household numbers from the bag one-by-one. Participation rates where high; yet, if a selected household refused to participate, another number was drawn until a willing household agreed to participate. The procedure continued until at least 15 households were selected (30 households in one village, 26016). For villages smaller than 15 households, all households were included. In all study villages, the final sample ranged from 9–30 households.

The study catchment consisted of small villages surrounding the Gola Rainforest, which is located in the post-conflict region bordering Sierra Leone and Liberia (Figures S1–S2 in File S1). A summary of the demographic characteristics and health variables of the study area is provided in the Tables S1–S2 in File S1. Friendship ties were elicited by asking household heads to “*Please give the full names of eight close friends that do not live in your household and that you would feel comfortable to either turn to for advice, ask for an interest-free loan, or ask for help with harvest without paying (only feeding). Please indicate if the person named lives in the village.*” Directed edges between pairs of households were generated if any member of the receiving household was named. When a household head named more than one friend from a particular household, these multiple edges were treated as one edge. Friends named within the same household as the respondent (self-loops) were ignored in the analysis.

To allow for reciprocation of a connection, only the households interviewed were used to construct the village networks. Furthermore, only households that either named a connection within our sample, or were named by a household within our sample were included in the network. Network summary statistics are presented in Table S3 in File S1. The nodes (N = 83) represent households and the edges (N = 124) represent friendship connections between them (Figure 1).

Interviewed households included in the derived networks were compared to interviewed households that were not used to construct the networks and were found to be balanced across a wide range of socio-economic variables except for the number of years the household has been in the village, agricultural occupation, social status, and belonging to the village 26007 (Tables S4–S5 in File S1). We account for this variation in sample selection by including these differences in extended models presented below to show that our findings uphold. To access the study data and code, please refer to the following supplementary information: Study Data S1, and Regressions S1.

### In-degree and betweenness variables

The directed network parameters of betweenness and in-degree were calculated and visualized using the Network Analyzer Plugin from Cytoscape version 2.8.3. We analyze subnetworks where the namers and named fall within the same sample. Both in-degree and betweenness depend on the number of nodes included in the derived network. The subnetworks result in a small number of nodes with many connections (high in-degree) and a small number of nodes with high connectivity (high-betweenness). We examined these properties, which are found in complete named networks [16], to identify trends across seven villages. Further, there is not a clearly defined one-unit increase of betweenness that is comparable to a one-unit increase of in-degree, which is the addition of one friend. Accordingly, we focused on the significance and direction of the coefficients of betweenness and in-degree to explain the associations found in this paper. We find that in-degree and betweenness are correlated, but are not perfectly collinear (Table S6 in File S1), which further supports that each centrality indicator independently explains different health outcomes.

### Statistical Analysis

**Figure 2:**
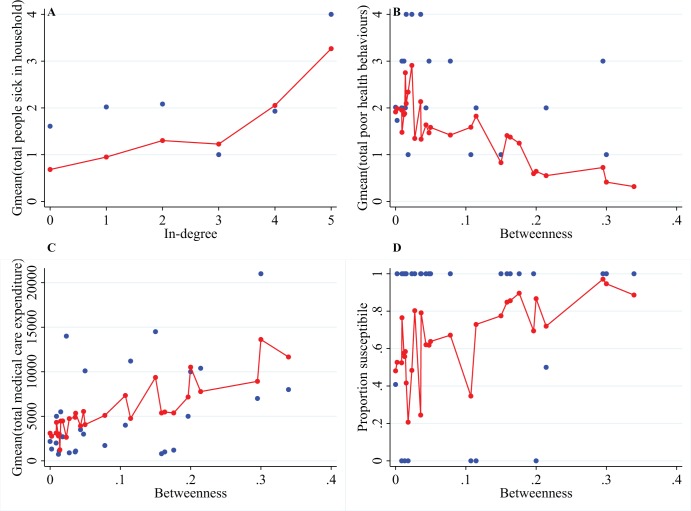
Significant centrality predictor in regressions on health outcomes. Blue dots are raw values and connected red dots are predicted values from general linear models. The geometric mean is plotted (Gmean), as multiple households have the same in-degree or betweenness value. All unique values for in-degree and betweenness are plotted. All regressions (N = 83) controlled for village-level fixed effects, not shown. For full regression results, see Table 1. **A)** In-degree is positively related to the self-reported morbidity indicator of total people sick in the household. In-degree coeff. 0.308 (p<0.05); Betweenness coeff. −0.581 (p>0.10). Negative binomial model. **B)** Betweenness is a measure of preventative health and negatively related to poor health behaviours. In-degree coeff. 0.034 (p>0.10); Betweenness coeff. −5.107 (p<0.01). Poission model. **C)** Betweeness positively predicts medical care expenditure. In-degree coeff. 398.84 Liberian dollars (LBD) (p>0.10); Betweenness coeff. 23,084.77 LBD (∼296.34 USD) (p<0.05). Gaussian model. **D)** Betweenness is an indicator of household head susceptibility to health influences from other villagers. In-degree coeff. −0.106 (p>0.10); Betweenness coeff. −7.117 (p<0.05). Binomial probit model.

Statistical analyses are conducted using Stata version 12.1. The regressions presented in Tables 1–2 and Figure 2, use general linear models with iterated least squares maximization and village-level fixed effects. Below, we discuss the relative importance of in-degree and betweenness for various health outcomes in a comparative analysis of regression models containing both variables.

**Table 1:** Betweenness and in-degree explain different aspects of health.

	(1A) Dependent variable: Total people sick in household			(1B) Dependent variable: Total people sick in household			(2) Dependent variable: Poor preventative health			(3) Dependent variable: Expenditures on drugs and hospital in past year			(4) Dependent variable: Health decisions influenced by other village members		
Explanatory variables	Coeff.	S.E.	p	IRR^a^	S.E.	p	Coeff.	S.E.	p	Coeff.	S.E.	p	Coeff.	S.E.	p
Betweenness	–0.581	2.447	0.812	0.560	1.369	0.812	–5.107**	1.921	0.008	23084.773*	9189.404	0.012	7.117*	2.933	0.015
In-degree	0.308*	0.137	0.025	1.360*	0.186	0.025	0.034	0.073	0.638	398.847	474.001	0.400	–0.106	0.131	0.420
*Village*															
13111	1.305*	0.653	0.046	3.689*	2.411	0.046	0.045	0.405	0.911	–1477.694	2256.778	0.513	–1.546*	0.684	0.024
13233	0.056	0.634	0.930	1.057	0.670	0.930	0.190	0.319	0.551	333.716	1915.353	0.862	–0.695	0.539	0.197
13245	–0.009	0.668	0.989	0.991	0.662	0.989	0.469	0.322	0.145	–441.597	2070.206	0.831	–1.497*	0.616	0.015
13247	–0.130	0.677	0.848	0.878	0.595	0.848	0.647	0.333	0.052	–3191.745	2119.459	0.132	–0.544	0.615	0.376
26007	1.095	0.707	0.121	2.990	2.113	0.121	0.108	0.455	0.812	1392.003	2484.751	0.575	–0.863	0.725	0.234
26016	0.352	0.524	0.502	1.422	0.746	0.502	0.243	0.277	0.381	–1591.255	1644.273	0.333	–0.552	0.470	0.240
Constant	–0.699	0.485	0.150	0.497	0.241	0.150	0.402	0.252	0.110	3709.068*	1461.369	0.011	0.763	0.429	0.075
N	83			83			83			83			83		

*p<0.05.

**p<0.01.

***<0.001.

a Incident rate ratio.

Panels (1A–B) Negative binomial, Panel (2) Poisson, Panel (3) Gaussian, and Panel (4) Binomial probit.

**Table 2:** Medical care and betweenness relation with self-reported health covariate.

	Dependent variable: Expenditures on drugs and hospital in past year		
Explanatory variables	Coeff.	S.E.	p
Betweenness	22890.199**	8652.674	0.008
In-degree	–1.152	463.110	0.998
Total people sick in past month	1191.798**	368.336	0.001
*Village*			
13111	–3356.593	2202.831	0.128
13233	199.533	1803.915	0.912
13245	–438.605	1949.243	0.822
13247	–3052.940	1996.080	0.126
26007	–706.153	2427.769	0.771
26016	–2018.970	1553.831	0.194
Constant	3316.840*	1381.310	0.016
N	83		

*p<0.05.

** p<0.01.

***<0.001.

GLM model was used with the Gaussian family.

## Results

### In-degree as a self-reported health indicator

In-degree positively predicts the number of people sick in a household (Table 1 Panel 1A and Figure 2A). Households with high in-degree (many close friends) report a higher number of total people sick in their home during the previous month than households with few or no incoming friendship links. The difference in the logs of the expected number of sick people changes by 0.308 (p<0.05) with each additional incoming friendship connection (Table 1 Panel 1A). In terms of the incidence rate ratio, with each additional connection (in-degree), the number of people sick increases by 1.360 (p<0.05, Table 1 Panel 1B). This finding is expected as in-degree is an indicator of popularity and the more popular an individual is the more likely they are to be ill.[22] By contrast, we do not observe a significant relation with betweenness and the total people sick in a household (coefficient (coeff.) −0.581, p>0.05, Table 1 Panel 1A). Additionally, there is village-level variation in health status. Living in village 13111 significantly contributes to the number of people sick in a household (coeff. 1.305, p<0.05, Table 1 Panel 1A).

### Betweenness as a health behaviour measure

Households with high betweenness avoid poor health behaviours and actively spend income on medical care. Poor health behaviours were measured by asking household heads if they conduct one of the following six behaviours at least once per week: drinking alcohol, smoking, burning cooking fuel indoors, entering freshwater bodies, open defecation, and sleeping outside. Betweenness results in a decrease (coeff. −5.107, p<0.01; coeff. is per unit betweenness–See Methods) of the log of expected count of poor preventative health behaviours that a household head engages in (Table 1 Panel 2 and Figure 2B). By contrast, in-degree is only weakly and insignificantly related to poor preventative health (coeff. 0.034, p>0.05). As the household head is the decision-maker of the family, their behaviours set an example for other household members. In rural Liberia, behavioural influence [25] is important for disease control as only 15.85% (13/82) of the study households had access to private latrines.

Households of high betweenness are not only cautious with respect to preventative health behaviours, but also actively seek formal medical care (Table 1 Panel 3 and Figure 2C). Medical care expenditure is defined as the total amount spent on drugs and hospital stays over the year preceding the study. We examine medical care separately from poor health behaviours, as expenditure on formal care is a distinct type of behaviour when compared to preventative health (Tables S7–S8 in File S1). These two behavioural indicators are neither collinear (VIF 1.00 for poor health behaviour and VIF 1.23 for medical care) nor correlated (Spearman coeff. 0.044, p>0.05). Betweenness is associated with a health expenditure increase (coeff. 23,085 Liberian Dollars (LD) or 296.34 USD per unit betweenness, p<0.05) in the yearly purchases of drugs and hospital payments. Again, in-degree is weakly and insignificantly related to not only the behavioural variable of poor preventative health, but also medical care expenditures (coeff. 399 LD, p>0.05).

Further, in Table 2, we control for the total number of people sick in a household. An additional person ill in the household increases yearly medical expenditures (coeff. 1,192 LD, p<0.01). Including the total people ill in a household does not affect our main result. Betweenness remains significant and positive (coeff. 22,890 LD, p<0.01). As expenditures on medical care have zero or positive values, we additionally construct Tobit models to corroborate the robustness of the betweenness and medical care relationship (Tables S9–S10 in File S1).

### Betweenness as an indicator for susceptibility to good health behaviours

In our study, betweenness explains susceptibility to positive health behaviours rather than influence, as found in other social network contexts.[19] In our survey, we ask, “*Is your decision to seek treatment or to engage in preventative health care behaviours affected by what other villagers are doing?*” As can be seen in Panel 4 of Table 1 and Figure 2D, betweenness increases the probability of susceptibility to the health behaviours of other people in the village (coeff. 7.117, p<0.05) whereas in-degree does not (coeff. −0.106, p>0.05). Additionally, village 13111 (coeff. −1.546, p<0.05) and village 13245 (coeff. −1.497, p<0.05) carry a lower probability of behavioural susceptibility of individuals.

Importantly, receptiveness to the actions of other villages is an indicator of engaging in good health behaviours. Susceptibility is positively correlated to good health behaviours with a Spearman coefficient of 0.283 (p<0.01, N = 83) and negatively, but not significantly, related to poor preventative health, with a weak Spearman coefficient of −0.018 (p>0.05, N = 83). Good health behaviour is defined as a count variable of the number of good behaviours that a household head engages in on a weekly basis including: hand-washing, use of public latrine, use of private latrine, boiling water, sleeping under a bed net, and taking packaged medicine.

### Extended models of network centrality and health with socio-economic covariates

Thus far, we presented models of in-degree and betweenness for self-reported physical health and actual health behaviours using only a minimal set of regressors. Understanding that a large number of variables for a small number of observations limit the degrees of freedom and model leverage, we also construct extended models (Table 3). The additional covariates are not perfectly collinear (Table S11 in File S1). In addition to the village-level fixed effects, the extended models incorporate agricultural occupation, years of residence in a village, and social status. Further, we include a wealth indicator of home quality to control for the impact of household wealth on the health of household members.

**Table 3:** Extended models of in-degree and betweenness for health.

Explanatory variables	(1A) Dependent variable: Total people sick in household			(1B) Dependent variable: Total people sick in household			(2) Dependent variable: Poor preventative health			(3) Dependent variable: Expenditures on drugs and hospital in past year			(4) Dependent variable: Health decisions influenced by other village members		
	Coeff.	S.E.	p.	IRR^a^	S.E.	p.	Coeff.	S.E.	p.	Coeff.	S.E.	p.	Coeff.	S.E.	p.
Betweenness	0.840	2.520	0.739	2.317	5.838	0.739	–4.897*	1.953	0.012	23317.543*	9172.032	0.011	6.813*	2.987	0.023
In-degree	0.297*	0.149	0.045	1.346	0.200	0.045	0.032	0.079	0.688	86.680	491.800	0.860	–0.062	0.140	0.658
Years in village	–0.028	0.017	0.100	0.972	0.017	0.100	–0.006	0.009	0.473	15.637	51.771	0.763	0.003	0.015	0.859
Agriculture	–0.212	0.405	0.600	0.809	0.327	0.600	–0.059	0.210	0.777	–1381.190	1253.283	0.270	0.254	0.365	0.488
Social status	0.011	0.342	0.975	1.011	0.346	0.975	0.022	0.194	0.909	2975.192**	1113.394	0.008	–0.147	0.338	0.663
Home quality score	–0.045	0.094	0.637	0.956	0.090	0.637	–0.027	0.055	0.626	165.977	318.196	0.602	–0.002	0.093	0.986
*Village*															
13111	1.293	0.678	0.057	3.643	2.471	0.057	0.086	0.418	0.838	–1562.047	2266.573	0.491	–1.539*	0.700	0.028
13233	0.341	0.652	0.601	1.407	0.917	0.601	0.265	0.328	0.420	–194.028	1944.527	0.921	–0.713	0.561	0.204
13245	0.121	0.703	0.863	1.129	0.793	0.863	0.503	0.336	0.134	624.011	2092.434	0.766	–1.621*	0.645	0.012
13247	0.094	0.701	0.893	1.099	0.771	0.893	0.702*	0.348	0.044	–3065.157	2195.864	0.163	–0.732	0.649	0.259
26007	1.119	0.744	0.132	3.062	2.277	0.132	0.140	0.465	0.762	1066.114	2544.107	0.675	–0.870	0.757	0.250
26016	1.031	0.630	0.102	2.803	1.766	0.102	0.419	0.333	0.209	–1807.954	1968.455	0.358	–0.609	0.577	0.291
Constant	–0.350	0.617	0.570	0.705	0.435	0.570	0.552	0.333	0.098	2059.261	1909.818	0.281	0.738	0.570	0.195
N	81			81			81			81			81		

*p<0.05.

**p<0.01.

***p<0.001.

a Incident rate ratio.

Panel (1A–B) Negative binomial, Panel (2) Poisson, Panel (3) Gaussian, and Panel (4) Binomial probit.

The additional covariates are defined as follows. Agricultural occupation is a binary variable indicating whether the main occupation of the household head was in agriculture. Social status is a binary variable and is positive if at least one household member has at least one important leadership position in the community including: elder council member, youth leader, women’s leader, societal head, village chief, tribal authority, or a mining chairman. Years in village is a count variable of the total years since the household has settled in the current village. The home quality score is a count indicator with range 0 to 18. Households were asked to indicate all materials used for any part of the home, such as, “*Does your home have a tarpaulin roof?*” Binary indicators were recorded and converted to scores. The floor, walls, and roof of the home are rated from 1–3 and added together to form the home quality score. The materials are provided in order of low to high quality. The floor materials are earth, wood, and concrete. The wall materials are mud and sticks, zinc, and cement. The roof materials are straw/thatch, tarpaulin, and zinc. For descriptive statistics of the covariates, see Tables S3–S4 in File S1.

With the additional covariates, we show that the network parameters of in-degree and betweenness still significantly explain self-reported health and health behaviour. In Panel 1A of Table 3, the log of the expected number of people ill changes by 0.297 (p<0.05) with each connection gained by a household when named by another household as a close friend. In other words, the incidence rate or number of new people ill in a household increases by 1.346 (p<0.05) for each new household connection (Table 3 Panel 1B). We do not observe a significant relation with betweenness and total people sick in the household (coeff. 0.840, p>0.05). In Panel 2 (Table 3), a one-unit increase in betweenness results in a 4.897 (p<0.05) decrease in the log of the number of poor preventative health behaviours. By contrast, in-degree is only weakly and insignificantly related to poor preventative health (coeff. 0.032, p>0.05). Additionally, belonging to village 13247 increases the log of the expected number of poor preventative health behaviours by 0.702 (p<0.05). Furthermore, in Panel 3 (Table 3), a one-unit increase in the betweenness of a household is associated with an expenditure increase of 23,317.54 LD (p<0.01) for medical care. Also, having a household member with high social status increases medical expenditures by 2,975.19 LD (p<0.01). Lastly, in Panel 4 (Table 3), a one-unit increase in betweenness increases the probability that an individual is influenced by the health actions of other villagers by a factor of 6.813 (p<0.05). In-degree is insignificantly (coeff. −0.062, p>0.05) related to health behaviour of other individuals. Individuals belonging to village 13111 (coeff. −1.539, p<0.05) and village 13245 (coeff. −1.621, p<0.05) are less likely to be influenced by other village members.

## Discussion

Social networks exhibit similar structures across developed and developing countries.[26] In this study, we ask whether social network analysis can inform healthcare provision in the developing world, and more specifically whether network centrality indicators can be used to identify self-reported health, the presence of good or bad health behaviours, and the response to positive social influence. Using data from highly remote and isolated villages in Liberia, we show that health status and health behaviour of an individual can be explained by the in-degree and betweenness of households in village friendship networks. We find that in-degree is a significant predictor of self-reported physical health, complementing past studies that show a positive correlation between in-degree and infection rates in a developed country context.[22] We also find that the heads of households with high betweenness avoid poor health behaviours and are more susceptible to social influence for good preventative health. In-degree explains exposure to environmental factors whereas betweenness explains the susceptibility to influence.

In rural areas of the developing world, access to healthcare is scarce, therefore, it is important to be able to identify those who need medical help the most, to encourage good health behaviours, and to utilize positive social influence in order to reduce morbidity in these areas. Our findings show that social network analysis can offer an alternative set of observable indicators and provide a better understanding as to why these challenges persist. In the context of our study villages in Liberia, social networks reveal that the sickest households are neither likely to engage in preventative health nor likely to be susceptible to positive health influences from the community.

### Data Availability Statement

All authors provide full access to the data used to perform the analysis in “Social network analysis predicts health behaviors and self-reported health in African villages.” Due to the sensitive nature of the data, i.e. the involvement of human participants, identification numbers in place of household names are provided to comply with ethical requirements. Further, as social status and household variables are included and can be backtracked to participants; village names were replaced with identifiers. The data are available within the Supporting Information files. The first sheet of the file contains all the regression data. The second sheet of the excel file contains the household linkages in long form. All predictor variable names are the same to those used in the main paper; these definitions can be found in the manuscript text. The dependent variable names are shortened versions of the descriptions used in the main-text. Accordingly, explanations of dependent variables also are provided in the script used for regressions. The annotated code used to execute the regressions in the statistical analysis software, Stata, is provided as a plain text file.

## Supporting Information

File S1 **Appendix containing two figures and eleven tables.** Figure S1, Map of study villages. Figure S2, Zoomed in map of study villages. Table S1, Summary statistics of study population for count and continuous variables. Table S2, Summary statistics of study population for binary variables. Table S3, Network summary statistics. Table S4, Two-tailed, two-sample t-tests for selection biases of households included in networks. Table S5, Chi-squared tests for sample biases of households included in networks. Table S6, Collinearity diagnostics of in-degree and betweenness. Table S7, Collinearity diagnostics of dependent health variables. Table S8, Spearman correlation of dependent health variables. Table S9, Medical care and network centrality. Table S10, Medical care and network centrality with self-reported health covariate. Table S11, Collinearity tests for covariates in extended models.
(DOCX)

Regressions S1 **Stata regression code.**
(TXT)

Study Data S1 **Excel file of data used in this paper.**
(XLS)

## References

[1] Murray CJ (2012) Disability-adjusted life years (DALYs) for 291 diseases and injuries in 21 regions, 1990–2010: a systematic analysis for the Global Burden of Disease Study 2010. Lancet 380: 2197–2223. 23245608 10.1016/S0140-6736(12)61689-4

[2] Vos T (2012) Years lived with disability (YLD) for 1160 sequelae of 289 diseases and injuries 1990–2010: a systematic analysis for the Global Burden of Disease Study 2010. Lancet 380: 2163–2196. 23245607 10.1016/S0140-6736(12)61729-2 PMC6350784

[3] Salomon JA (2012) Healthy life expectancy for 187 countries, 1990–2010: a systematic analysis for the Global Burden of Disease Study 2010. Lancet 380: 2144–2162. 23245606 10.1016/S0140-6736(12)61690-0

[4] Wang H (2012) Age-specific and sex-specific mortality in 187 countries, 1970–2010: a systematic analysis for the Global Burden of Disease Study 2010. Lancet 380: 2071–2094. 23245603 10.1016/S0140-6736(12)61719-X

[5] Lozano R (2012) Global and regional mortality from 235 causes of death for 20 age groups in 1990 and 2010: a systematic analysis for the Global Burden of Disease Study 2010. Lancet 380: 2095–2128. 23245604 10.1016/S0140-6736(12)61728-0 PMC10790329

[6] Pruss-Ustun A, Corvalan C (2006) Preventing disease through healthy environments: towards an estimate of the environmental burden of disease. (WHO), p 16.

[7] Hotez P (2009) Mass drug administration and integrated control for the world’s high-prevalence neglected tropical diseases. Clin Pharmacol Ther 85: 659–664. 19322166 10.1038/clpt.2009.16

[8] Fewtrell L, Kaufmann RB, Kay D, Enanoria W, Haller L, et al (2005) Water, sanitation, and hygiene interventions to reduce diarrhoea in less developed countries: a systematic review and meta-analysis. Lancet Infect Dis 5: 42–52. 15620560 10.1016/S1473-3099(04)01253-8

[9] Chami G, Molyneux D, Kontoleon A, Dunne D (2013) Exploring network theory for mass drug administration. Trends Parasitol 29: 370–379. 23742966 10.1016/j.pt.2013.04.005

[10] Aral S, Walker D (2012) Identifying Influential and Susceptible Members of Social Networks. Science 337: 337–341. 22722253 10.1126/science.1215842

[11] Szell M, Thurner S (2013) How women organize social networks different from men. Sci Rep 3: 01214. 10.1038/srep01214 PMC3566601 23393616

[12] Bramoullé Y, Djebbari H, Fortin B (2009) Identification of peer effects through social networks. J Econometrics 150: 41–55.

[13] Smith K, Christakis N (2008) Social networks and health. Annu Rev Sociol 34: 405–429.

[14] Kitsak M, Gallos LK, Havlin S, Liljeros F, Muchnik L, et al (2010) Identification of influential spreaders. Nat Phys 6: 888–893.

[15] Bond R, Fariss CJ, Jones JJ, Kramer ADI, Marlow C, et al (2012) A 61-million-person experiment in social influence and political mobilization. Nature 489: 295–298. 22972300 10.1038/nature11421 PMC3834737

[16] Albert R, Barabási AL (2002) Statistical mechanics of complex networks. Rev Mod Phys 74: 50.

[17] Newman M (2010) Measures and metrics. Networks: an introduction (Oxford University Press Inc., New York), pp 168–169, 185–193.

[18] Newman M (2004) A measure of betweenness centrality based on random walks. Soc Net 27: 39–54.

[19] Goh KI, Oh E, Kahng B, Kim D (2003) Betweenness centrality correlation in social networks. Phys Rev E 67: 017101. 10.1103/PhysRevE.67.017101 12636633

[20] Christakis N, Fowler J (2008) The collective dynamics of smoking in a large social network. New Engl J Med 358: 2249–2258. 18499567 10.1056/NEJMsa0706154 PMC2822344

[21] Christakis N, Fowler J (2007) The spread of obesity in a large social network over 32 years. New Engl J Med 357: 370–379. 17652652 10.1056/NEJMsa066082

[22] Christakis N, Fowler J (2010) Social network sensors for early detection of contagious outbreaks. PLoS ONE 5: e12948. 20856792 10.1371/journal.pone.0012948 PMC2939797

[23] Liljeros F, Edling C, Amaral L, Stanley E, Aberg Y (2001) The web of human sexual contacts. Nature 411: 907–908. 11418846 10.1038/35082140

[24] Liberia Institute of Statistics and Geo-Information Services (LISGIS), Ministry of Health and Social Welfare, National AIDS Control Program, Macro International Inc. 2008 (2008) Liberia demographic and health survey 2007. (Monrovia, Liberia).

[25] Kruk M, Rockers P, Varpilah S, Macauley R (2011) Which doctor? Determinants of utilization of formal and informal health care in postconflict Liberia. Med Care 49: 585–591. 21422954 10.1097/MLR.0b013e31820f0dd4

[26] Apicella C, Marlowe F, Fowler J, Christakis N (2012) Social networks and cooperation in hunter-gatherers. Nature 481: 497–501. 22281599 10.1038/nature10736 PMC3340565

