# Supplementary material for: Social Network Analysis Predicts Health Behaviours and Self-Reported Health in African Villages
Source: PLoS One. 2014 Jul 29;9(7):e103500. doi: 10.1371/journal.pone.0103500 (PMC4114748; doi:10.1371/journal.pone.0103500)
Supplement: File S1 — Appendix containing two figures and eleven tables. Figure S1, Map of study villages. Figure S2, Zoomed in map of study villages. Table S1, Summary statistics of study population for count and continuous variables. Table S2, Summary statistics of study population for binary variables. Table S3, Network summary statistics. Table S4, Two-tailed, two-sample t-tests for selection biases of households included in networks. Table S5, Chi-squared tests for sample biases of households included in networks. Table S6, Collinearity diagnostics of in-degree and betweenness. Table S7, Collinearity diagnostics of dependent health variables. Table S8, Spearman correlation of dependent health variables. Table S9, Medical care and network centrality. Table S10, Medical care and network centrality with self-reported health covariate. Table S11, Collinearity tests for covariates in extended models. (DOCX) [file pone.0103500.s001.docx]

**Supplementary Information Appendix for:**

Social network analysis predicts health behaviours and self-reported health in African villages

Goylette F. Chami, Sebastian E. Ahnert, Maarten J. Voors, Andreas A. Kontoleon

Table of Contents

Figure S1: Map of study villages 2

Figure S2: Zoomed in map of study villages 3

Table S1: Summary statistics of study population for count and continuous variables 4

Table S2: Summary statistics of study population for binary variables 4

Table S3: Network summary statistics 5

Table S4: Two-tailed, two-sample t-tests for selection biases of households included in networks 6

Table S5: Chi-squared tests for sample biases of households included in networks 7

Table S6: Collinearity diagnostics of in-degree and betweenness 7

Table S7: Collinearity diagnostics of dependent health variables 8

Table S8: Spearman correlation of dependent health variables 8

Table S9: Medical care and network centrality 9

Table S10: Medical care and network centrality with self-reported health covariate 9

Table S11: Collinearity tests for covariates in extended models 10

References 10

### Figure S1: Map of study villages


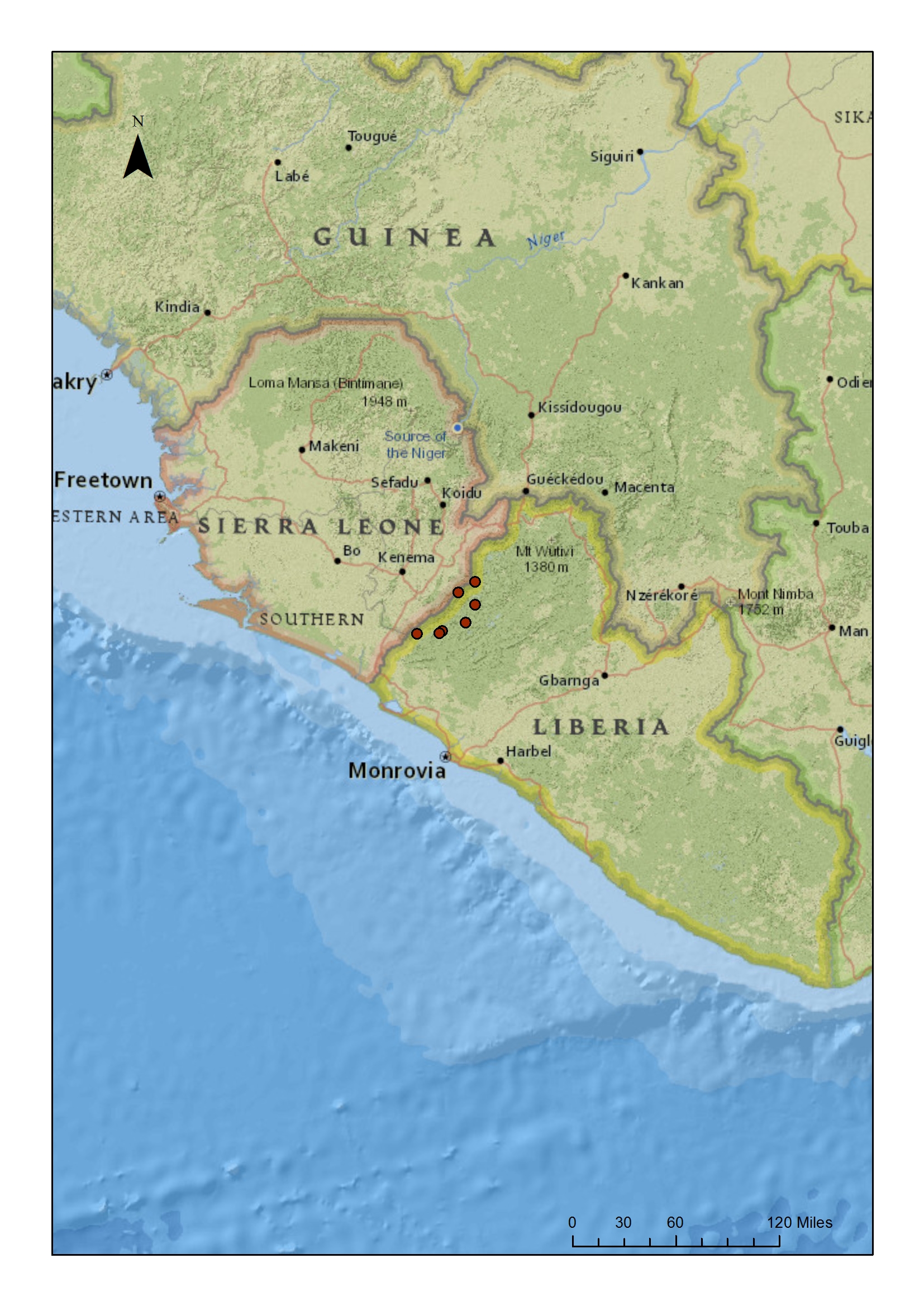


### Figure S2: Zoomed in map of study villages

**
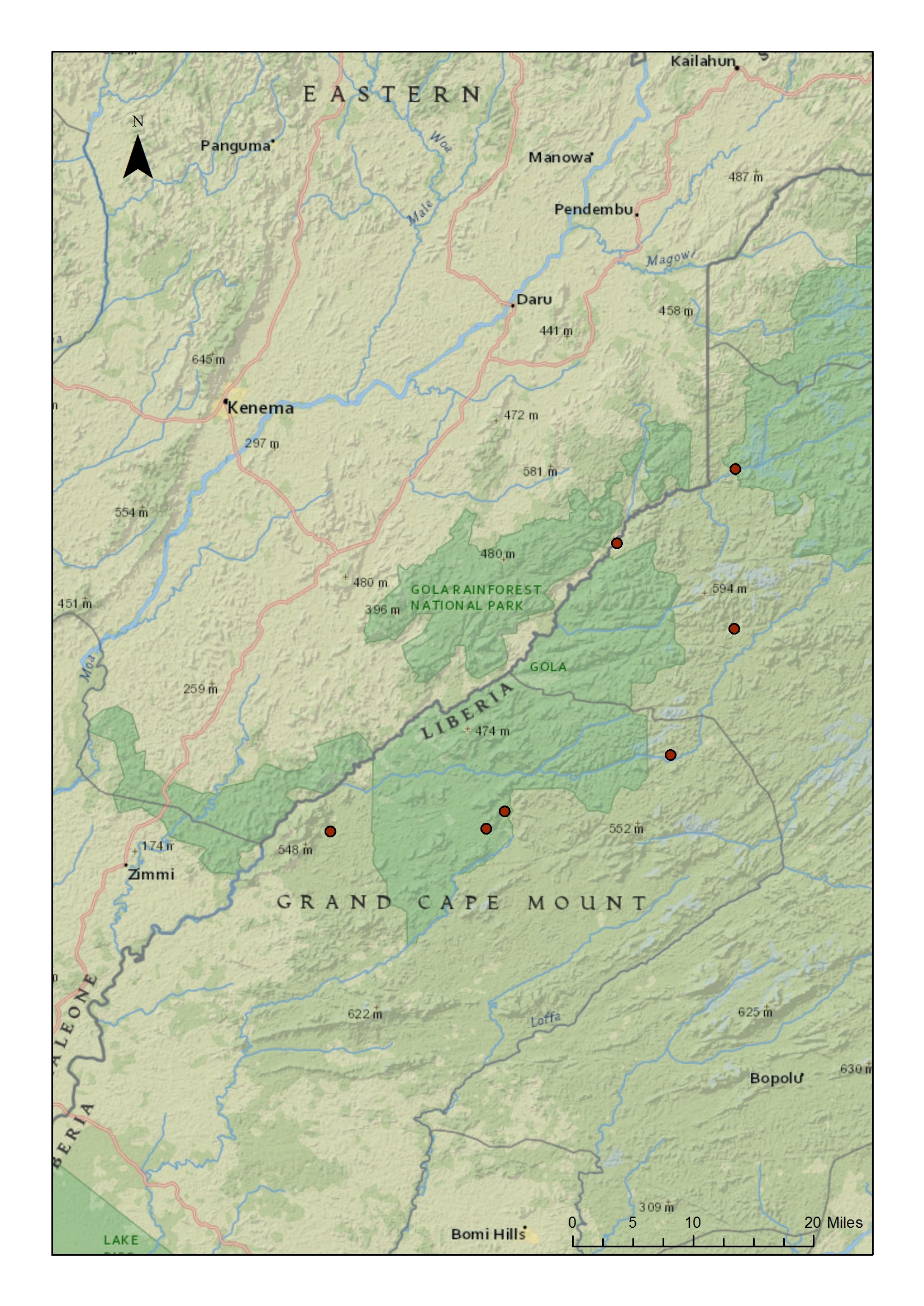
**

### Table S1: Summary statistics of study population for count and continuous variables

| **Count or continuous variables** |  |  |  |  |  |
| --- | --- | --- | --- | --- | --- |
| **Variable** | **Obs.** | **Mean** | **SD** | **Min** | **Max** |
| Betweenness | 83 | 0.038 | 0.078 | 0 | 0.339 |
| In-degree | 83 | 1.446 | 1.318 | 0 | 5 |
| Total people sick in household | 83 | 1.229 | 1.517 | 0 | 8 |
| Expenditure on drugs and hospital in past year (medical care) | 83 | 4250.952 | 4984.716 | 0 | 23000 |
| Poor preventative health | 83 | 1.759 | 1.245 | 0 | 4 |
| Years in village | 81 | 15.506 | 14.081 | 0 | 60 |
| Home quality score | 83 | 4.867 | 1.999 | 2 | 10 |

### Table S2: Summary statistics of study population for binary variables

| **Binary variables** |  |  |  |
| --- | --- | --- | --- |
| **Variable** | **Obs.** | **Frequency** | **Percent** |
| Health decisions influenced by other village members | 83 | 46 | 55.42 |
| Agriculture | 83 | 25 | 30.12 |
| Social status | 83 | 42 | 50.6 |
| *Village* 13111 | 83 | 9 | 10.84 |
| 13233 | 83 | 12 | 14.46 |
| 13245 | 83 | 9 | 10.84 |
| 13247 | 83 | 10 | 12.05 |
| 26007 | 83 | 6 | 7.23 |
| 26016 | 83 | 25 | 30.12 |
| 26036 | 83 | 12 | 14.46 |

### Table S3: Network summary statistics

| **Village** | **Total nodes (Households)*** | **Total Edges**** | **Total nodes/ total physical homes** | **Total nodes/ total households** | **Total nodes/ households interviewed** | **Total physical homes** | **Total households** | **Households interviewed** | **Total friends named that were inside the village** | **Number of friends named matched to an interviewed household** | **Friends matched/total inside village friends** |
| --- | --- | --- | --- | --- | --- | --- | --- | --- | --- | --- | --- |
| 13111 | 9 | 11 | 0.643 | 0.205 | 0.6 | 14 | 44 | 15 | 32 | 12 | 0.375 |
| 13233 | 12 | 11 | 0.3 | 0.2 | 0.8 | 40 | 60 | 15 | 41 | 16 | 0.390 |
| 13245 | 9 | 16 | 1.286 | 0.692 | 1 | 7 | 13 | 9 | 27 | 16 | 0.593 |
| 13247 | 10 | 23 | 0.714 | 0.589 | 1 | 14 | 17 | 10 | 46 | 23 | 0.5 |
| 26007 | 6 | 10 | 0.102 | 0.071 | 0.4 | 59 | 85 | 15 | 39 | 10 | 0.256 |
| 26016 | 25 | 37 | 0.5 | 0.202 | 0.833 | 50 | 124 | 30 | 110 | 41 | 0.373 |
| 26036 | 12 | 16 | 0.429 | 0.308 | 0.8 | 28 | 39 | 15 | 57 | 16 | 0.281 |
| Total or *Average* | 83 | 124 | *0.568* | *0.323* | *0.776* | 212 | 382 | 109 | 352 | *19.143* | *0.395* |
| *There are 26 households that were interviewed that do not appear in the friendship networks. | | | | | |  |  |  |  |  |  |
| *There may be fewer edges included in the network then friends matched to interviewed households because multiple edges between the same pair of households were treated as 1 edge. | | | | | | | | | | | |

The inclusion of interviewed households varies from 40-83%. Additionally, the village networks exhibit low reciprocity. Named household heads are not likely to name other household heads in return; only 19.35% or 24/124 (12 pairs) of ties was reciprocated.

### Table S4: Two-tailed, two-sample t-tests for selection biases of households included in networks

|  |  | **Household is included in the friendship network** | |  |  |  |
| --- | --- | --- | --- | --- | --- | --- |
| **Variable** |  | **no** | **yes** | ***t*** | ***p*** | ***df*** |
| War shocks | mean | 3.385 | 5.450 |  |  |  |
|  | SD | 3.556 | 6.019 | -1.655 | 0.101 | 104 |
|  | obs. | 26 | 80 |  |  |  |
| Civil conflicts | mean | 0.423 | 0.337 | 0.589 | 0.557 | 107 |
|  | SD | 0.703 | 0.630 |  |  |  |
|  | obs. | 26 | 83 |  |  |  |
| Years in village | mean | 4.885 | 15.506 | -3.724 | 0.0003 | 105 |
|  | SD | 6.173 | 14.081 |  |  |  |
|  | obs. | 26 | 81 |  |  |  |
| Household size | mean | 3.962 | 4.867 | -1.910 | 0.059 | 107 |
|  | SD | 1.843 | 2.185 |  |  |  |
|  | obs. | 26 | 83 |  |  |  |
| Average household age | mean | 23.235 | 25.128 | -0.846 | 0.3995 | 107 |
|  | SD | 8.681 | 10.317 |  |  |  |
|  | obs. | 26 | 83 |  |  |  |
| Average household education | mean | 2.377 | 2.305 | 0.207 | 0.836 | 106 |
|  | SD | 1.410 | 1.563 |  |  |  |
|  | obs. | 25 | 83 |  |  |  |
| Income | mean | 32938.460 | 49049.000 | -0.601 | 0.549 | 107 |
|  | SD | 53336.350 | 132922.500 |  |  |  |
|  | obs. | 26 | 83 |  |  |  |
| Home quality score | mean | 5.385 | 4.867 | 1.118 | 0.266 | 107 |
|  | SD | 2.246 | 1.999 |  |  |  |
|  | obs. | 26 | 83 |  |  |  |

### Table S5: Chi-squared tests for sample biases of households included in networks

| **Variable** | **chi-squared** | **p** | **df** |
| --- | --- | --- | --- |
| Agriculture | 7.5247 | 0.006 | 1 |
| Mining | 0.0144 | 0.905 | 1 |
| Social status | 10.067 | 0.002 | 1 |
| *Village* |  |  |  |
| 13111 | 2.4967 | 0.114 | 1 |
| 13233 | 0.1422 | 0.706 | 1 |
| 13245 | 3.4489 | 0.063 | 1 |
| 13247 | 12.512 | 0.000 | 1 |
| 26007 | 1.1770 | 0.278 | 1 |
| 26036 | 0.1422 | 0.706 | 1 |
| Electricity | 0.1969 | 0.657 | 1 |
| Religion | 1.6304 | 0.202 | 1 |
| *Tribe* |  |  |  |
| Mende | 0.1774 | 0.674 | 1 |
| Gola | 0.2652 | 0.607 | 1 |
| Vai | 1.6416 | 0.200 | 1 |
| Gio | 0.4854 | 0.486 | 1 |
| Kpelle | 0.4854 | 0.486 | 1 |
| Mano | 0.6382 | 0.424 | 1 |
| Gbandi | 0.1526 | 0.696 | 1 |
| Believes sickness caused by witchcraft | 0.0030 | 0.956 | 1 |

### Table S6: Collinearity diagnostics of in-degree and betweenness

|  |  | **SQRT** |  | **R-** |  | **Conditional** |
| --- | --- | --- | --- | --- | --- | --- |
| **Variable** | **VIF** | **VIF** | **Tolerance** | **Squared** | **Eigenvalue** | **Index** |
| In-degree | 1.46 | 1.21 | 0.6837 | 0.3163 | 3.0665 | 1 |
| Betweenness | 1.91 | 1.38 | 0.5242 | 0.4758 | 1.2871 | 1.5435 |
| **Mean In-degree and betweenness VIF = 1.685** | | | |  |  |  |

High degree nodes (range = 0 - 5) are moderately correlated with high betweenness nodes (range = 0 - 0.339) with a Spearman correlation coefficient of 0.580 (p<0.001, N=83). This correlation is expected as nodes of high in-degree are relatively more connected in the network and there is a greater likelihood of a shortest path passing through them ^1^ ^2^. However, with high betweenness, a household does not have to have many friends (high in-degree) to be well placed in the network ^3^. In Table S6, the variance inflation factor (VIF) is small for in-degree (1.46) and betweenness (1.91), which is below the conventional limit of 10 VIF ^4^. Here, the square roots of the VIFs explain the inflation of the standard error that is caused by the correlation of in-degree with betweenness. Considering the square root of the VIFs, the standard errors of in-degree and betweenness are only inflated by a factor of 1.21 and 1.38, respectively. Accordingly, we are able to separately measure how variations in in-degree and betweenness affect health in our models.

### Table S7: Collinearity diagnostics of dependent health variables

| **Variable** | **VIF** | **Sqrt VIF** | **Tolerance** | **R-Squared** | **Eigenvalue** | **Conditional Index** |
| --- | --- | --- | --- | --- | --- | --- |
| Total people sick in household | 1.24 | 1.11 | 0.8044 | 0.1956 | 3.4493 | 1 |
| Health decisions influenced by other village members | 1.03 | 1.01 | 0.9754 | 0.0246 | 0.6913 | 2.2337 |
| Poor preventative health | 1 | 1 | 0.9983 | 0.0017 | 0.4045 | 2.9201 |
| Expenditures on drugs and hospital in past year | 1.23 | 1.11 | 0.8143 | 0.1857 | 0.3236 | 3.2649 |
| Mean | VIF | 1.12 |  |  |  |  |

### Table S8: Spearman correlation of dependent health variables

|  | **Total people sick in household** | **Expenditures on drugs and hospital in past year** | **Health decisions influenced by other village members** | **Poor preventative health** |
| --- | --- | --- | --- | --- |
| Total people sick in household | 1 |  |  |  |
| Expenditures on drugs and hospital in past year | 0.4403*** | 1 |  |  |
|  | (0) |  |  |  |
| Health decisions influenced by other village members | -0.2286* | 0.0263 | 1 |  |
|  | (0.0376) | (0.8132) |  |  |
| Poor preventative health | 0.044 | -0.0268 | -0.0089 | 1 |
|  | (0.6925) | (0.8101) | (0.9367) |  |
| p-value in parentheses | * p<0.05 ** p<0.01 ***p<0.001 | |  |  |

### Table S9: Medical care and network centrality

|  | **Dependent variable: Expenditures on drugs and hospital in past year** | | |
| --- | --- | --- | --- |
| **Explanatory variables** | Coef. | S.E. | p |
| Betweenness | 26167.15** | 9234.062 | 0.006 |
| In-degree | 484.4768 | 475.2672 | 0.311 |
| *Village* |  |  |  |
| 13111 | -2122.656 | 2276.07 | 0.354 |
| 13233 | 375.714 | 1902.386 | 0.844 |
| 13245 | -506.6318 | 2056.273 | 0.806 |
| 13247 | -4312.624 | 2175.066 | 0.051 |
| 26007 | 626.2353 | 2508.779 | 0.804 |
| 26016 | -1860.01 | 1639.355 | 0.26 |
| Constant | 3576.344* | 1453.745 | 0.016 |
| Sigma | 4646.703 | 377.5671 |  |
| N | 83 |  |  |
| * p<0.05 ** p<0.01 ***<0.001 | |  |  |
| Tobit model with lower limit of 0.  6 left-censored observations at medical_care<=0  77 uncensored observations | | | |

### Table S10: Medical care and network centrality with self-reported health covariate

|  | **Dependent variable: Expenditures on drugs and hospital in past year** | | |
| --- | --- | --- | --- |
| **Explanatory variables** | Coef. | S.E. | p |
| Betweenness | 26370.82** | 8629.893 | 0.003 |
| In-degree | 30.42426 | 459.3314 | 0.947 |
| Total people sick in past month | 1339.56*** | 366.9778 | 0.000 |
| *Village* |  |  |  |
| 13111 | -4369.54 | 2229.625 | 0.054 |
| 13233 | 225.2522 | 1770.037 | 0.899 |
| 13245 | -505.433 | 1912.742 | 0.792 |
| 13247 | -4177.589* | 2029.475 | 0.043 |
| 26007 | -1854.213 | 2456.98 | 0.453 |
| 26016 | -2338.167 | 1531.695 | 0.131 |
| Constant | 3137.543* | 1358.175 | 0.024 |
| Sigma | 4322.356 | 349.908 |  |
| N | 83 |  |  |
| * p<0.05 ** p<0.01 ***<0.001 | |  |  |
| Tobit model with lower limit of 0.  6 left-censored observations at medical_care<=0  77 uncensored observations | | | |

### Table S11: Collinearity tests for covariates in extended models

| **Variable** | **VIF** | **Sqrt VIF** | **Tolerance** | **R-squared** | **Eigenvalue** | **Conditional Index** |
| --- | --- | --- | --- | --- | --- | --- |
| Betweenness | 2.94 | 1.72 | 0.3399 | 0.6601 | 3.2529 | 1 |
| In-degree | 2.29 | 1.51 | 0.4368 | 0.5632 | 2.8655 | 1.0655 |
| Agriculture | 1.6 | 1.26 | 0.6254 | 0.3746 | 2.3232 | 1.1833 |
| Social status | 1.86 | 1.37 | 0.5366 | 0.4634 | 1.7356 | 1.369 |
| Years in village | 2.62 | 1.62 | 0.3815 | 0.6185 | 1.4084 | 1.5198 |
| Home quality score | 1.69 | 1.3 | 0.5928 | 0.4072 | 0.0958 | 5.8267 |

### References

1. Barabási A-L & Albert R. (1999) Emergence of scaling in random networks. *Science*;**286**:509-512.

2. Goh K-I, Kahng B, & Kim D. (2001) Universal behavior of load distribution in scale-free networks. *Phys. Rev. Lett.*;**87**:278701.

3. Goh K-I, Oh E, Kahng B, et al. (2003) Betweenness centrality correlation in social networks. *Phys. Rev. E* ;**67**:017101.

4. Nester J, Wasserman W, & Kutner MH (1989) Applied linear regression models (Irwin, Homewood, IL).
